# Adult predation shapes the evolution of swimming performance in guppies (*Poecilia reticulata*)

**DOI:** 10.1098/rsbl.2025.0139

**Published:** 2025-06-25

**Authors:** Mingfang Yang, Hannah De Waele, Arjan P. Palstra, Alexander Kotrschal

**Affiliations:** ^1^ Behavioural Ecology Group, Wageningen University & Research, Wageningen, Gelderland, The Netherlands; ^2^ Animal Breeding and Genomics Group, Wageningen University & Research, Wageningen, Gelderland, The Netherlands

**Keywords:** swimming performance, *U*_crit_, predation pressure, guppy, evolution, artificial selection

## Abstract

Predation pressure plays an important role in shaping animal behaviour and physiology, driving prey species to evolve stronger escape strategies. Swimming performance is a key trait for many aquatic organisms to evade predation. It is therefore intuitive that increased predation pressure should select for faster swimming abilities when outswimming predators is a viable option for prey. However, experimental evidence allowing for a causal link between predation and the evolution of swimming performance is currently lacking. Here, we used artificial selection lines of guppies (*Poecilia reticulata*) based on predation survival to test the evolutionary relationship between predation pressure and swimming speed. We used a swim tunnel with incremental increase in water flow to test critical swimming speed. Our results show that predation-line females, but not males, outperformed those of the control-lines in critical swimming speed. We also found that in predation-line females the variance in critical swimming speed was reduced in comparison with control-line females, which is congruent with directional selection against slow swimming genotypes. This study provides experimental evidence for the evolutionary role of predation pressure in enhancing swimming performance and shaping behavioural adaptations in prey species.

## 1. Introduction

Predation is a major selective force driving the evolution of survival-related traits in prey species [1–5]. Prey adapts to predation pressure in manifold ways, including morphological changes, such as alterations in body shape in perch (*Perca fluviatilis*) [6] and behavioural changes, such as decreased sensitivity and response frequencies in hard clams (*Mercenaria mercenaria*) [7], improved predator avoidance in wall lizards (*Podarcis muralis*) [8] and increased activity in poecilid fish (*Podarcis muralis*) [9]. Understanding how predation pressure shapes these traits is central to evolutionary biology, as it provides insights into the mechanisms underlying evolutionary changes in prey species. Although many studies have explored the broad impacts of predation on prey, establishing a causal relationship between predation pressure and prey evolution remains challenging as in ‘natural’ experiments increased or decreased predation pressure typically concurs with other factors such as predator species [10], habitat size [11] and food abundance [12], which limit the interpretations of wild situations to correlative findings. Even in laboratory experiments, an obvious limitation when comparing experimental populations with and without a predator, is that the impact of living with predators differs across life stages [13–15]. New artificial selection lines focusing on survival under predation pressure during a brief adult period provide an unprecedented opportunity to address these limitations. One such experiment has recently been conducted using the Trinidadian guppies (*Poecilia reticulata*). These fish were artificially selected for adult survival under predation pressure [16]. By comparing the offspring of populations that have been subjected to three generations of such predation selection to the offspring of control lines, we can causally investigate trait evolution [17].

Swimming performance plays a crucial role in avoiding predators in aquatic environments. Fish exhibit several categories of swimming performance, such as sustained, prolonged and burst swimming, though definitions vary across studies [18,19]. Although these swimming types have different emphases, fish generally trade-off endurance and speed [20]. Guppies often face sudden predator attacks in the wild, so they typically need to respond with short bursts of swimming often followed by a swim through open water to escape predators [21], suggesting that swimming performance is important for guppies to avoid predators. Critical swimming speed is widely used to assess swimming performance in fish by incrementally increasing swimming speed at prescribed intervals until fish fatigue [18,22,23]. As this measurement is crucial for evaluating the physiological and ecological adaptability of fish in response to predation pressure [14,24,25], we here test the critical swimming speed in the predation selected guppy lines.

To measure swimming performance under conditions that are ecologically relevant for guppies, we use increasing water velocity over a relatively short period until guppies are exhausted. Predation selection on swimming performance leads to an intuitive hypothesis that guppies from high predation pressure environments have been selected for better swimming performance. By comparing the swimming performance of guppies from predation and control lines, we investigate whether predation-selection fish outperform control lines in swimming performance and whether there is directional selection under predation pressure.

## 2. Material and methods

### (a) Experimental fish

The fish used in this study were laboratory lines of Trinidadian guppies (*Poecilia reticulata*) that were artificially selected for survival under predation pressure [16,26]. Briefly, the predation lines were created by cohabiting adults (males and females separately) with their natural predator (*Crenicichla alta,* now taxonomically classified as *Saxatilia frenata*) for a few weeks and pairing the survivors with the survivors of the opposite sex, while the control lines were created by pairing individuals that were kept in the same tanks, but safe from being eaten. The tanks were designed to mimic a guppy stream with different depths (2–40 cm water depth) and their large size allowed for natural predator–prey interactions, similar to those in natural ponds. The selection procedure was repeated for three generations, with three replicates per line [16]. The fish used for this experiment were the offspring (approximately 6−9 months old) of the last selected generation; 213 virgin females (standard length (SL): 2.36 ± 0.23 cm) and 215 males (SL: 1.78 ± 0.14 cm) balanced over selection lines and replicates.

### (b) Swim tunnel system

We assessed swimming performance using a 30 l swim tunnel system (Loligo, Viborg, Denmark) with a swimming area of (length × width × height): 46 × 14 × 14 cm. Flow velocities that corresponded with voltage values of the motor that drove the propellor to generate the flow were measured by Agbeti *et al*. [27].

### (c) Swimming performance measurement

Six fish of the same sex were placed in the swim tunnel for a 3 min acclimation period (no water flow). We chose a swimming regime to match the ecology of guppies in the wild, where they typically escape predators by short bursts, sometimes followed by pursuits through open water [21]. Although guppies may not typically reach exhaustion in the wild, variation in the critical swimming speed can still reflect underlying physiological traits shaped by selection, particularly in environments with predation risk [24,25]. After the acclimation, the voltage was initially set to 0.4 V (flow velocity: 12.51 cm s^−1^, corresponding to about 5 SL s^−1^ for females and 7 SL s^−1^ for males) and was subsequently increased in 0.1 V increments, corresponding to an average additional flow velocity increase of 4.56 cm s^−1^ (about 2 SL s^−1^ for females, 2.5 SL s^−1^ for males) every 30 s. Six fish were tested together in the swim tunnel. Each fish was tested until it fatigued and could no longer maintain its position in the swim section, touching the rear net for over 3 s without being able to remove itself [28]. Afterward, the fish was removed one at a time and placed in a separate tank for recovery. The exhausted fish was photographed for body size measurements. Swimming time of the fish was recorded. The water was exchanged daily. The temperature was maintained at 25°C throughout the experiment. For swimming performance, we calculated critical swimming speed (*U*_crit_) using the following formula:


$$U_{crit}\left(cm\;s^{-1}\right)=V_{m}+\left(\left(T_{s}/T\right)\times V_{i}\right),$$

where *V*_m_ (cm s^−1^) is the highest velocity completed before exhaustion, *T*_s_ (s) is the duration swum at the final flow velocity before exhaustion, *T* ( = 30 s) is the fixed time increment and *V*_i_ (cm s^−1^) is the flow velocity increment.

### (d) Statistics

All data were examined for normality and homogeneity of variance using Shapiro–Wilk test and Bartlett test, respectively. If the data did not meet the assumptions of normality or homogeneity of variance, we used a generalized linear mixed model (GLMM) to account for non-normality. When the data met both assumptions, we used a linear mixed model (LMM), which is appropriate for normally distributed data. A generalized linear mixed model was first used to assess difference in *U*_crit_ between the females and males, with sex, selection line and selection line replicates as fixed effects, standard length as a covariate and group as a random factor. The data for females and males were then analysed separately to ensure the applicability and interpretation of the model for each sex. To test the difference in standard length of guppies between the selection lines, we used a linear mixed model with selection line and selection line replicates as a fixed factor and group as a random factor. For each sex separately, we used analogous generalized linear mixed models to assess difference in *U*_crit_ between the predation and control lines, with selection line and selection line replicates as fixed effects, standard length as a covariate and group as a random factor. We used a Fligner–Killeen test and *F-*test to test for potential variance differences in *U*_crit_ and body size between the selection line, respectively. As we found significant variance difference in females, we adjusted the variance structure in the female GLMM, using a heteroscedastic model, ensuring a more accurate fit to the data. All statistical analyses were conducted using RStudio (2023.6.1.524), and a probability level of *p* < 0.05 was considered as being significant.

## 3. Result

**Figure 1.**
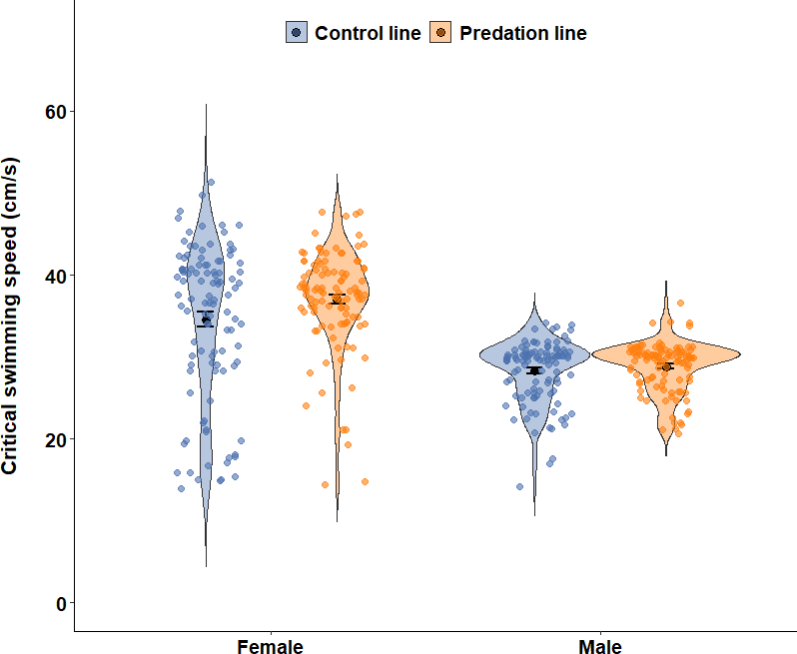
The critical swimming speed of male and female guppies that were subjected to three generations of artificial selection for adult predation survival (orange) and corresponding control lines (blue). The black dots show the mean critical swimming speed for each line, with short black error bars representing the standard error (SE). Violin plots show the distribution of the data.

Fish of the selection lines did not differ in body size (standard length, females: *t* = −0.022, *p* = 0.982; males: *t* = −0.005, *p* = 0.996) (electronic supplementary material, table S1), nor in variance in body size (females: *F* = 0.954, *p* = 0.808; males: *F* = 0.874, *p* = 0.487), and females swam significantly faster than males (*t* = −7.898, *p* < 0.001) (electronic supplementary material, table S2). We found that females from the predation lines had a higher *U*_crit_ than those from the control lines (table 1, *p* = 0.033; control line: 34.54 ± 9.46 cm s^−1^, predation line: 37.01 ± 6.19 cm s^−1^), whereas no such difference was observed for males (table 2, *p* = 0.251; control line: 28.28 ± 3.79 cm s^−1^, predation line: 28.84 ± 3.03 cm s^−1^) (figure 1). Additionally, the variance in *U*_crit_ was significantly lower in predation-line females compared with control-line females (*χ*² = 16.377, *p* < 0.001). Further, standardized variance in *U*_crit_ was also lower in the predation-line females (0.028) than in the control-line females (0.075), indicating reduced relative variability. In males, there was no difference in variance between predation and control lines (*χ*² = 1.790, *p* = 0.181), with standardized variance values of 0.011 (predation line) and 0.018 (control line). We also found that replicate significantly affected female swimming performance (table 1, *p* = 0.008), indicating that differences in replicate conditions (e.g. tank environment, handling) could influence swimming performance. For males, standard length also influenced swimming performance (table 2, *p* = 0.034).

**Table 1.** Outcomes of statistical models for the critical swimming speed (*U*_crit_) of females that were subjected to three generations of artificial selection for adult predation survival versus their control lines. Number of individuals = 213 (predation line: 107, control line: 106).

	estimate	std error	*z-*value	*p-*value
fixed effects				
(intercept)	3.659	0.158	23.164	<0.001
selection line (P)	0.072	0.034	2.126	0.033
standard length	−0.095	0.067	−1.407	0.159
replicate	0.052	0.020	2.639	0.008
random effects	variance	s.d.	
group (intercept)	<0.001	<0.001		

**Table 2.** Outcomes of statistical models for the critical swimming speed (*U*_crit_) of males that were subjected to three generations of artificial selection for adult predation survival versus their control lines. Number of individuals = 215 (predation line: 108, control line: 107).

	estimate	std error	*t-*value	*p-*value
fixed effects				
(intercept)	3.605	0.112	32.307	<0.001
selection line (P)	0.020	0.017	1.149	0.251
standard length	−0.130	0.061	−2.12	0.034
replicate	−0.016	0.010	−1.536	0.124
random effects	variance	s.d.		
group (intercept)	<0.001	0.006		
residual	<0.001	0.117		

## 4. Discussion

Here we tested the effect of three generations of intense adult predation pressure on the evolution of swimming performance and found that females become better swimmers. While results for females align with guppy ecology and our predictions, the absence of an effect of predation selection on male swimming performance is less clear.

Our findings show that females evolved better swimming performance under predation, which is unsurprising as we used a natural predator that often hunts guppies in pursuits [10,29] and relatively large naturalistic ‘ponds’ with deep and shallow water area to perform the predation selection [16]. Individuals with relatively better swimming abilities will outswim slower individuals and thereby be caught less, favouring non-random selection on swimming speed in females, hence the evolution of swimming performance. Our previous study also found that females from the predation lines appeared more ‘streamlined’, with relatively smaller body area, which could have contributed to improved swimming efficiency [16]. This is consistent with the idea that predation drives adaptive changes in traits directly linked to escape performance [30,31]. For example, high-predation females exhibit faster burst-swimming performance, including higher acceleration, velocity and greater escape distances compared with low-predation females [32,33]. How did the predation-line females evolve on average better swimming performance than the control lines? Visual inspection of figure 1 suggests that rather than increasing maximal swimming performance, this process was driven by selective removal of the relatively slowest individuals. In line, we found a reduced standardized variance in swimming performance in predation-line compared with control females. This is consistent with what is often observed in such relatively short-term artificial selection experiments, where differences typically materialize via selection on standing genetic variation and not via de novo mutations [28,34]. We conclude that predation is a powerful selective force driving rapid evolutionary changes in prey traits that favour escape ability. Our findings for females are in line with previous studies on swimming performance of guppies from wild populations under different predation pressures [14,35]. However, due to the fact that we isolated predation as the only selective force differing between predation and control lines, we here provide the first direct and causal evidence for its role in shaping swimming performance.

Predation seems to select for better swimming performance in female guppies. However, in males we found no such effect. One explanation is that sexual selection may outweigh predation selection in shaping male swimming performance. In general, the effects of predation selection on different traits in an organism often result in compromises between competing selective pressures [30]. Male guppies face strong sexual selection for traits like bright coloration, elaborate fins and courtship displays, which may trade-off with escape performance driven by predation [14,31,36]. In line, data from the field also seems to suggest that predation does not exert strong selection on male swimming performance, as male guppies did not exhibit clear differences based on predation level in endurance swimming test [14]. Congruently, some other studies found no strong correlation between predation regime and aerobic or burst performance in male guppies [37,38]. In addition, a study showed that males and females respond differently to acute predation cues, which supports the idea that males may experience weaker predation selection and show less evolutionary change in swimming performance [39]. However, we suggest that it is too early to conclude that predation does not impact male swimming performance. Unlike the larger females, who may be followed for longer by predators and so require more efficient swimming to evade, males may be selected for rapid bursts rather than sustained high-speed swimming for mating success [14]. This aspect of male swimming performance needs to be tested further. While we found no difference in critical swimming speed between male selection lines, standard length was a significant factor in male swimming performance, suggesting that body size plays an important role. Even more, such aspects of swimming performance should be tested as we recently found that predation selected males develop shorter tails and gonopodia in comparison with males from control lines [16]. We had attributed this to adaptation for better swimming performance for predator evasion. While our current findings of no difference in swimming performance may seem to contradict such reasoning, predation selection may not increase the integrated measure of speed and endurance we tested here, but rather impact the evolution of swimming via selecting for increase in for instance top speed in escape bursts, or reaction time.

We also observed that male guppies had lower swim endurance than females, regardless of the selection lines. This could be due to several non-mutually exclusive reasons. For one, female guppies are larger than male guppies, which helps them to swim faster, as larger individuals typically have greater swimming performance [40]. Additionally, physiological differences, such as muscle efficiency or metabolic rate, might contribute to the observed higher swimming speed in females. Sex-specific differences in muscle utilization during swimming has been shown to contribute to variation in swimming speeds between male and female guppies [41].

In conclusion, our findings highlight the critical role of predation pressure in driving rapid evolutionary changes in swimming performance. In addition, our results showed sex-specific responses to predation selection, which supports the idea that predation pressure does not act uniformly across sexes.

## Data Availability

All data and R code are available from the Dryad Digital Repository [42]. Supplementary material is available online [43].
